# Commentary: “How Much is that Player in the Window? The One with the Early Birthday?” Relative Age Influences the Value of the Best Soccer Players, but Not the Best Businesspeople

**DOI:** 10.3389/fpsyg.2016.00620

**Published:** 2016-04-28

**Authors:** Florian Loffing

**Affiliations:** Institute of Sports and Sports Science, University of KasselKassel, Germany

**Keywords:** relative age effects, birth date, statistics, outliers, bias

Furley et al. (2016) recently reported that among the 100 most valuable soccer players, but not the 100 richest billionaires, individuals born in the first compared to the second half of the year were overrepresented (60 vs. 40%) and had higher estimated monetary value (EMV).

The idea of testing whether birthdate-related selection cut-off dates are associated with players' EMV is attractive. Implications for people advocating equal chances of high sporting achievement might be suggested, provided that evidence is substantive. Here I show that the data do not support the authors' interpretation that “relative age influences the value of the best soccer players.”

Instead of categorizing players into birth halves and thereby losing potentially meaningful information (examples of own work that can be legitimatelly criticized in this respect: Loffing et al., 2010; Schorer et al., 2015), correlational analysis could have been used to test for an association between month of birth and EMV. Neither, parametric (*r* = −0.067, *p* = 0.509) nor non-parametric tests (e.g., τ_b_ = −0.004, *p* = 0.955) indicate that EMV decreases as players are born later in the year (Figure 1A)^1^.

**Figure 1:**
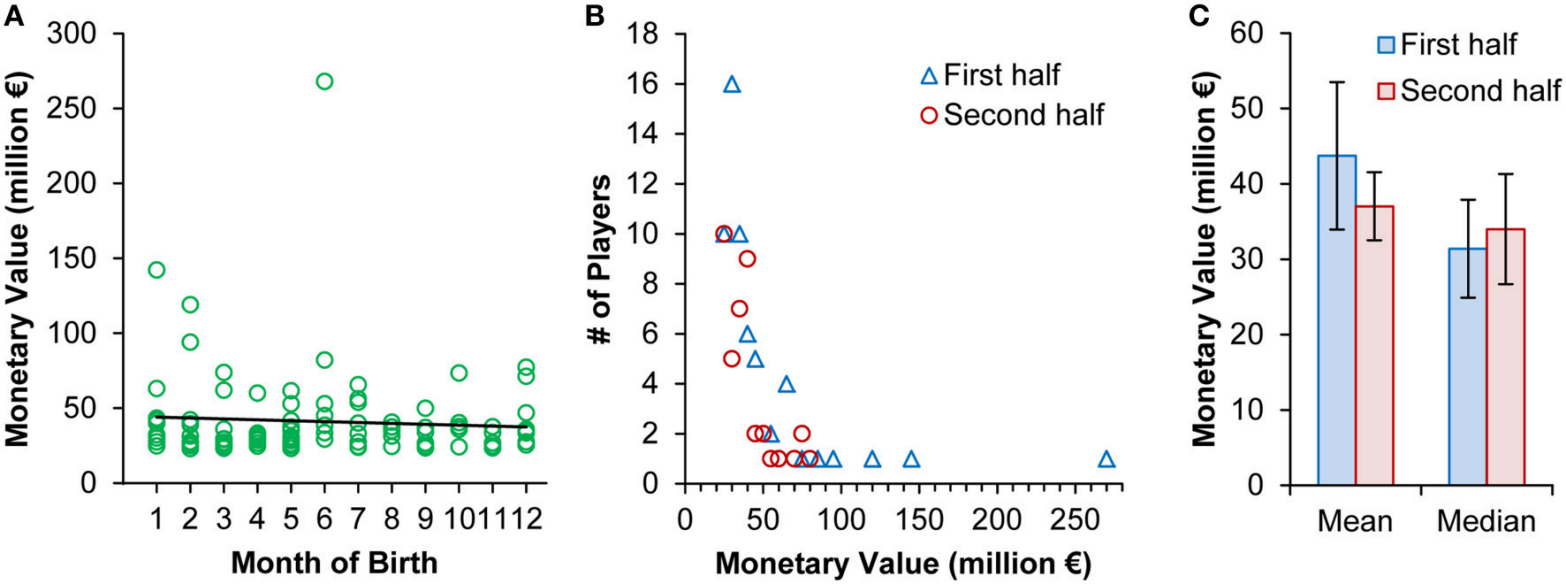
**(A)** Soccer players' estimated monetary value as a function of players' month of birth (1 = January, …, 12 = December). The solid line illustrates the linear relationship between the two variables (*r*^2^ = 0.004; *y*_EMV_ = −0.5946x_MoB_ + 44.52). **(B)** Frequency distribution of EMV in players born in the first vs. second half of the year based on a categorization of players' EMV in 5 million € intervals. **(C)** Mean (± 95% confidence intervals) and median (± median absolute deviation) estimated monetary value in players born in the first vs. second half of the year.

The authors' conclusion “that birthdates (…) can actually result in higher monetary value” (p. 2) is based on a biased *t*-statistic^2^. Eighty-two players have an EMV below 50 million €, whereas three players have an EMV above 100 million € (see Figure 1B). Lionel Messi (born on 24 June 1987) is the most valuable player (268.05 million €). Thus, among the most valuable players very few have very outstanding EMV. These “outliers” bias the *t*-statistic; e.g., there is a clear Lionel Messi effect. To illustrate, exclusion of Messi only, *t*_(97)_ = 0.696, *p* = 0.488, *d* = 0.14 (−0.26, 0.54), or assuming him to be born just 1 week later on 1 July 1987 (i.e., second half of the year), *t*_(98)_ = −0.432, *p* = 0.666, *d* = −0.09 (−0.48, 0.31), nullifies the authors' conclusion (e.g., see the range in 95% CIs for effect sizes calculated with Exploratory Software for Confidence Intervals; Cumming, 2012).

However, EMV are not distributed normally neither overall (e.g., Kolmogorov-Smirnov test: *Z*_(100)_ = 0.275, *p* < 0.001) nor within groups [first half: *Z*_(59)_ = 0.289, *p* < 0.001; second half: *Z*_(41)_ = 0.213, *p* < 0.001; Figure 1B]. To avoid that single cases bias interpretation (e.g., compare means and medians illustrated in Figure 1C), a non-parametric test like Mann-Whitney-U seems a reasonable alternative. With Lionel Messi included, no group difference is found, *U* = 1191, *z* = −0.130, *p* = 0.897, *r* = −0.013. Importantly, even if there were a meaningful EMV difference between players born in the first vs. second half of the year this would not indicate “influence” of relative age on EMV, but only suggest an association between the two variables at best.

Another concern relates to the comparison of the proportion of players born in the first vs. second half of the year against an equal distribution. Relative age seems relevant to selection into professional soccer as reflected in a skewed birth distribution in the population of professional soccer players (e.g., Musch and Hay, 1999; Cobley et al., 2008; Helsen et al., 2012; Schorer et al., 2015). If we take a specific look on, say, the 100 most valuable players and want to test whether being born in the first vs. second half of the year alters the chances of being among the top 100, these players' birth distribution can *a priori* be expected to be skewed. Therefore, it should be tested against soccer professionals' birth distribution (57.53 vs. 42.47%; big-5 European Leagues in the season 2015/2016; see Supplementary Material online for details), not against a “uniform” distribution in the general population. Doing so reveals no relevant effect, $\chi_{\left(1,N=100\right)}^{2}=$ 0.09, *p* = 0.77, *OR* = 1.06 (0.61, 1.86). While exemplified here, the critique on reference values may similarly apply to other relative age research in sports as well (see Delorme and Champely, 2015, for details).

“Taken together, (…) broad implications that need to be taken seriously by political decision makers” (Furley et al., 2016, p. 2) should be based on proper study design and statistical methods in whatever domain. The above concerns illustrate that the data considered by Furley et al. (2016) do not legitimate their conclusions. A commentary on the study's limitations was necessary, first, to point out the importance of data inspection and critical assessment of the impact of individual cases on statistics, and second, to not suppress the discussion of a potential “underdog”-effect, which suggests that, in the long run, players born relatively later in the year may even have an advantage, or no disadvantage at least, in adult elite sporting competition (e.g., Ashworth and Heyndels, 2007; Schorer et al., 2009; Gibbs et al., 2012). Given the latter aspect, a directed hypothesis in favor of players born earlier in the year seems not well-grounded and, therefore, here all tests are two-tailed (however, see Gibbs et al., 2015, for a critique on applying inferential statistics on such sort of data).

Furley et al. must be honored for making their dataset publicly available. This is an important step toward transparency in science, thereby hopefully facilitating reproducibility as well as evaluation of data analysis and interpretation pre (e.g., during peer review) and post publication (Drummond and Vowler, 2011; Open Science Collaboration, 2015). Regrettably, the dataset at hand illustrates that single birthdays may mess[i] up your statistics.

## Author contributions

FL re-analyzed the data, wrote the manuscript and approved the final, submitted version of the manuscript.

### Conflict of interest statement

The author declares that the research was conducted in the absence of any commercial or financial relationships that could be construed as a potential conflict of interest. The reviewer, HH, and handling Editor declared their shared affiliation, and the handling Editor states that the process nevertheless met the standards of a fair and objective review.

## Footnotes

^1^EMV is reported in Euro, not in Dollars as done by the authors, because the dataset provided online lists players' values in Euro. Use of either currency, however, does not alter the outcome of inferential statistics.

^2^There were also some errors in original data coding. James Rodriguez (12 July 1997, 65.65 million €) was categorized as being born in the first half of the year and EMV of Manuel Neuer (36 million €, 27 March 1987) and Mesut Özil (35.85 million €, 15 October 1988) were interchanged. Here, corrected values are used.
